# Time to Huddle: Initiating the Bedside Sepsis Huddle on an Acute Care Pediatric Unit

**DOI:** 10.1097/pq9.0000000000000061

**Published:** 2018-04-17

**Authors:** Tracy S. Lowerre, Jonathan A. Silverman, Jill M. McGehee

**Affiliations:** From the * Acute Care Pediatric Unit, Children’s Hospital of Richmond at VCU, Richmond, Va.; † Pediatric Emergency Department, Children’s Hospital of Richmond at VCU; ‡ Pediatric Intensive Care Unit, Children’s Hospital of Richmond at VCU, Richmond, Va.

**Keywords:** Sepsis, Huddle, Sepsis huddle, Pediatric, Screening

## Abstract

**Background:**

Rapid recognition and prompt treatment of sepsis within 1 hour may improve sepsis outcomes in children.

**Objectives:**

To develop and implement a sepsis screening tool and huddle process to improve early recognition and treatment of sepsis on an inpatient pediatric unit.

**Methods:**

Sepsis huddle implementation entailed house-wide education on early recognition of sepsis, antibiotic administration, and fluid bolus delivery methods. We used rapid Plan Do Study Act (PDSA) cycles to enhance documentation and communication among care providers during sepsis huddles. The team developed pocket cards for all team members to screen patients for sepsis based on Systemic Inflammatory Response Syndrome (SIRS) criteria (Goldstein, 2005). A paper huddle form was developed and adapted to ensure all the bundle elements were implemented within the 1 hour goal. We added a sepsis huddle section to the vital sign band of the electronic medical record. Standardized sepsis order sets ensured consistent care between the ED, inpatient units, and PICU.

**Results:**

Between April 24 and December 1, there were 112 huddles, 28 rapid response activations, and 14 immediate transfers to a higher level of care. The pediatric department met the goal of 70% of first dose STAT antibiotics administered within 1 hour. Our Vizient observed to expected sepsis mortality has dropped from 1.37 (FY 2016) to 0.99 (FY 2017, preliminary data). (Table 1)

**Table 1.**
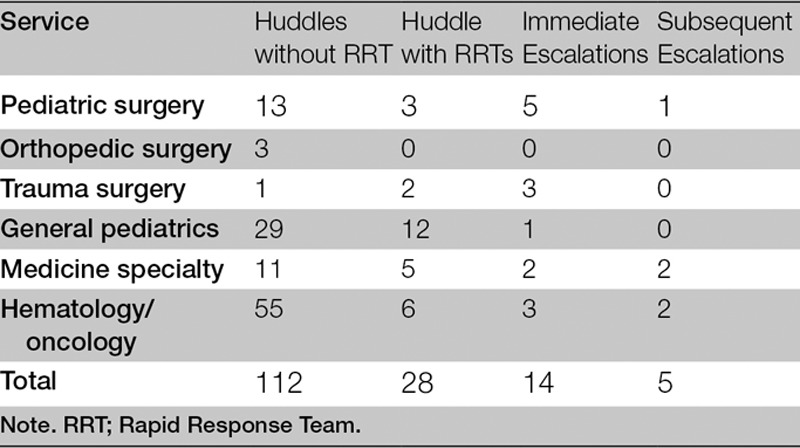
Huddles by Medical Team

